# HyRA-CXR: a hybrid residual–attention deep network for chest X-ray classification

**DOI:** 10.3389/frai.2026.1767330

**Published:** 2026-05-04

**Authors:** Ahmeed Suliman Farhan, Umar Manzoor, Ali Al-kubaisi, Ayman Jalil, Zeeshan Perveez, Ahmed B. Mohammd, Wadhah Zeyad Tareq

**Affiliations:** 1 Electronic Computer Center, University of Anbar, Ramadi, Iraq; 2 School of Architecture, Computing and Engineering, University of Wolverhampton, Wolverhampton, United Kingdom; 3 Computer Sciences and Information Technology, University of Anbar, Ramadi, Iraq; 4 Deptartment of Computer Engineering, Faculty of Engineering and Natural Sciences, Istinye University, Istanbul, Türkiye

**Keywords:** attention mechanisms, attention modules, chest X-ray, compact architecture, CXR, DenseNet121, dual attention mechanisms, Grad-CAM

## Abstract

Chest X-ray (CXR) interpretation is essential for diagnosing pulmonary diseases, yet manual reading remains slow and prone to human error, especially in high-volume or resource-limited settings. To address delayed diagnoses and improve clinical efficiency, this study introduces (HyRA-CXR), a hybrid residual–attention convolutional neural network for automated CXR classification. The proposed model integrates residual blocks to enhance gradient stability and dual attention mechanisms to focus on significant lung regions. Experiments are conducted on the publicly available Lung X-Ray Image dataset. Hyperparameters are optimized using KerasTuner as well as model evaluation is carried out using five-fold stratified cross-validation. HyRA-CXR achieved an average accuracy of 90.39%, outperforming DenseNet121 (89.38%) and Xception (89.12%) models. Also, the experimental results confirmed that both residual and attention modules contribute, as removing either reduced accuracy below 90%. Overall, the proposed model achieves competitive accuracy with maintaining a compact architecture (0.52M parameters), indicating its suitability for deployment in resource-constrained settings. Our code is publicly available at: https://github.com/Ahmeed-Suliman-Farhan/HyRA-CXR-A-Hybrid-Residual-Attention-Deep-Network-for-Chest-X-Ray-Classification.

## 1 Introduction

Chest radiography accounts for approximately 2 billion examinations annually, representing over 40% of all diagnostic imaging worldwide (Raoof et al., 2012). Yet manual interpretation remains diagnostically uncertain: inter-observer agreement among radiologists ranges from 0.40–0.70 across common thoracic pathologies (Rajpurkar et al., 2018), subtle abnormalities are frequently missed in high-volume settings (Hwang et al., 2019), and anatomical overlap obscures early-stage disease (Wang et al., 2017a). Clinical consequences are severe—pneumonia mortality increases 7% per hour without treatment (Kumar et al., 2006), and tuberculosis remains the world's leading infectious disease killer (World Health Organization, 2026). The global shortage of radiologists compounds these challenges, transforming AI-assisted diagnosis from theoretical curiosity to practical necessity.

Convolutional neural networks have achieved radiologist-level performance on specific CXR pathologies (Rajpurkar et al., 2018; Irvin et al., 2019), validating deep learning's capacity for end-to-end feature learning (LeCun et al., 2015). Despite these advances, conventional CNN architectures still face several limitations. Their fixed receptive fields make it difficult to capture both fine-scale texture patterns and broader spatial relationships within the image (Çallı et al., 2021). In addition, standard CNNs treat extracted features with similar importance, which may reduce their ability to focus on diagnostically relevant regions (Ouyang et al., 2021). To mitigate these problems, recent studies have also proposed hybrid models that integrate CNNs with attention mechanisms or Vision Transformers. For instance, CNN-ViT hybrid models like HyCoViT have achieved accuracy up to 98.81% (Naghash et al., 2025). However, these models require a significant number of parameters (over 85 million), which is computationally expensive and limits their applicability in point-of-care devices (Naghash et al., 2025). Moreover, additional stages in the attention module may also increase latency by up to 15–20 ms, which is undesirable for real-time applications (Ye et al., 2024). Another limitation is that these models may not generalize to other datasets, as previous studies have reported performance degradation by 12%–18% for unseen datasets without domain adaptation (Zech et al., 2018).

Recent reviews have also highlighted some of the methodological limitations in chest X-ray (CXR) image classification works. For example, 38% of existing works have used only a single split of data for training and testing, without employing other robust validation schemes such as cross-validation. In addition, 52% of existing works have failed to provide confidence intervals for their models, which is an important aspect in assessing model reliability. Furthermore, 19% of existing works have also shown extremely high accuracy rates (≥99%), which raises concerns of possible overfitting or using simple classification problems (Roberts et al., 2021). Another limitation is in hyperparameter tuning, with only 23% of existing works describing a systematic hyperparameter optimization process (Tiu et al., 2022). On the other hand, some of the latest works in hybrid CNN-Transformer models also report highly accuracy rates but are also extremely large in size, with some models having over 85 million parameters (Naghash et al., 2025). This is also an issue in these models, especially in medical systems. On the other hand, lightweight models such as MobileNetV3 are also proposed in some works but are found to have difficulty in maintaining high accuracy rates in multi-class settings (63.83%).

Although residual connections (He et al., 2016), which help stabilize gradient flow in deep networks, and attention mechanisms (Ouyang et al., 2021; Bhowal et al., 2021) have each shown benefits in chest X-ray (CXR) analysis, their combined use within a lightweight architecture has not been thoroughly evaluated. In particular, few studies examine this combination using rigorous experimental settings such as stratified cross-validation, systematic hyperparameter tuning, and detailed ablation analysis. Many existing works that integrate residual learning with attention modules rely on a single train–test split or provide limited analysis of individual components. As a result, it remains unclear whether both mechanisms contribute complementary benefits or whether the performance improvement is mainly driven by one of them.

To explore these issues, this study addresses the following research questions:

**RQ1:** Do residual connections and dual attention mechanisms provide complementary improvements in CXR classification performance, or does one component dominate the contribution of the other?
**RQ2:** Can a lightweight hybrid architecture that integrates residual learning with attention achieve competitive performance compared with established deep models when evaluated using stratified cross-validation and systematic hyperparameter optimization?
**RQ3:** Do visual explanation methods, including Grad-CAM and native attention maps, indicate that the proposed HyRA-CXR model focuses primarily on lung regions rather than irrelevant image cues (such as borders or embedded text) when generating predictions?

In this work, we introduce **Hy**brid **R**esidual-**A**ttention for **C**hest **X**-**R**ay classification (HyRA-CXR), a lightweight architecture designed to address the limitations identified in previous studies. Our contributions can be summarized as follows.

**(1) Architectural contribution:** we design a compact architecture that integrates lightweight residual blocks with dual attention mechanisms. Despite containing only 0.52M parameters, the proposed model achieves an accuracy of 90.39% ± 1.30% on a three-class CXR classification task. Under the same evaluation protocol, this performance exceeds that of several established architectures, including DenseNet121 (89.38%), Xception (89.12%), and MobileNetV3-Large (63.83%). Although some studies report higher absolute accuracies, these results are typically obtained under different experimental conditions, such as single train–test splits, binary classification settings, or datasets with visually distinct pathologies, which limits direct comparability.
**(2) Methodological contribution:** to ensure a robust evaluation, we adopt stratified five-fold cross-validation combined with systematic hyperparameter optimization using KerasTuner across 30 configurations. In addition, component-level ablation experiments are conducted to assess the role of each module. Removing the residual connections leads to a decrease of 0.72 percentage points in accuracy, while removing the attention modules results in a reduction of 0.63 percentage points. These results suggest that the two components provide complementary benefits when used together.
**(3) Interpretability contribution:** the predictions made by the model have been analyzed using Grad-CAM and native attention visualizations. From the explanation maps provided in the study, it is evident that HyRA-CXR is mainly focused on the lung regions of the images, whereas other unwanted regions of the images, such as borders or text, have not been considered.

The study indicates that with appropriate residual learning and attention mechanisms, it is possible to develop an effective model for classifying chest X-rays using fewer parameters than what is used in Vision Transformers.

## 2 Literature review

Over the past few years, deep learning has increasingly been used as the main approach for analyzing chest X-ray (CXR) images. Despite this rapid progress, previous studies show noticeable differences in several aspects, such as the structure of the neural networks used, the strategies adopted for training, the methods applied to evaluate performance, and the extent of clinical validation. For this reason, this section provides a brief overview of the most relevant developments in this area. It focuses on several key points: (i) architectural trends in CXR classification, (ii)Training Methodologies and Data Efficiency, (iii) Explainability, Clinical Validation, and Deployment, (iv) Performance Claims and Methodological Rigor, and (v) Open Challenges and Motivation for HyRA-CXR.

### 2.1 Architectural evolution in CXR classification

Early studies using convolutional neural networks (CNNs) demonstrated the feasibility of automated chest X-ray analysis by leveraging transfer learning from ImageNet-pretrained models (Wang et al., 2017b). These initial efforts showed that pretrained networks could be effectively adapted for multi-label thoracic disease classification on large-scale CXR datasets. Later investigations further highlighted the advantages of pretraining. For example, Mirugwe et al. (2025) reported a tuberculosis detection accuracy of 99.4% using a pretrained VGG16 architecture, and other comparative analyses have reported performance gains of approximately 4–7 percentage points when transfer learning is used instead of training models entirely from scratch (Gazda et al., 2021). Nevertheless, the usefulness of pretraining on natural image datasets for medical imaging tasks continues to be debated. One of the main concerns is the inherent domain gap: features learned from ImageNet are primarily optimized for recognizing everyday objects, whereas radiographic analysis requires sensitivity to subtle anatomical structures and pathological patterns.

To better capture diagnostically relevant information, later research explored the integration of attention mechanisms within CNN-based frameworks. These mechanisms were motivated by the observation that clinically significant regions in chest X-rays may appear in different spatial locations depending on the disease type. Ouyang et al. (2021) proposed a dual-attention architecture designed to model both channel-wise feature relevance and spatial dependencies, which resulted in notable performance improvements on the CheXpert dataset. Building on this idea, Guan et al. (2020) introduced attention-guided architectures capable of modeling bilateral anatomical relationships across lung regions. In another approach, Han et al. (2020) incorporated attention-based multiple instance learning, achieving an AUC of 94% for pneumonia detection.

Recent developments have explored hybrid architectures that integrate convolutional neural networks with Vision Transformers in a unified framework. In these models, CNN components are typically used to extract localized visual features, while Vision Transformers capture broader contextual relationships across the image. This design leverages the complementary characteristics of the two approaches: CNNs are well suited for identifying fine-grained textural details within restricted spatial regions, whereas ViTs employ self-attention mechanisms that enable modeling long-range dependencies across the entire image. Naghash et al. (2025) introduced HyCoViT achieving state-of-the-art 98.81% three-class accuracy, surpassing pure CNNs by 4.90 percentage points and pure transformers by 2.05 percentage points through custom CNN blocks paired with ViT self-attention. Their Dynamic Dropout algorithm—adaptively adjusting rates from 0.2 to 0.5 during training—combined with MixUp augmentation addresses data scarcity endemic to medical imaging. Kotei and Thirunavukarasu (2024) demonstrated that careful architectural design achieves both accuracy (99.38% tuberculosis detection) and extreme efficiency (6.9M parameters, 4.79 ms inference) through depth-wise separable convolutions. However, ViTs often underperform CNNs on small medical datasets unless heavily pretrained due to their lack of inductive biases (translation equivariance, locality), requiring larger training data to learn basic visual patterns that CNNs encode architecturally. These studies evaluated performance on different datasets with varying task difficulties—binary tuberculosis detection (visually distinct presentations) vs. multi-class pneumonia classification (overlapping radiographic features)—complicating direct architectural comparisons and raising questions about generalizability across clinical contexts.

Specialized components including scalable attention for multi-scale lesion detection (Lin et al., 2023) and residual connections for gradient stability (Narin et al., 2021) have demonstrated task-specific value. Transfer learning from ImageNet-pretrained models remains the dominant training paradigm for CXR classification. Kolonne et al. (2021) demonstrated that a MobileNetV2 architecture with modified top layers achieves 98.65% average accuracy on three-class CXR classification (Pneumonia, COVID-19, Normal) using five-fold cross-validation, while Fernando et al. (2022) extended this comparison across MobileNetV2, ResNet50, InceptionV3, and Xception under identical training conditions, with ResNet50 achieving 98.87% average accuracy and recall of 98.54%. However, both studies employed ImageNet pretraining and evaluated on datasets with COVID-19 as a distinct class rather than the Lung Opacity category used in the present work, where overlapping radiographic features between opacity and viral pneumonia present a more challenging discrimination task. These results nonetheless establish important transfer learning baselines against which from-scratch approaches can be contextualized. The proposed HyRA-CXR synthesizes these insights by integrating residual blocks with dual attention mechanisms, balancing gradient flow preservation through skip connections with adaptive feature refinement while avoiding full Vision Transformer computational overhead.

### 2.2 Training methodologies and data efficiency

One of the persistent difficulties in chest X-ray analysis is the relatively limited availability of labeled datasets, which often increases the risk of model overfitting during training. To address this limitation, Gazda et al. (2021) introduced a self-supervised contrastive learning framework for CXR analysis, where model encoders are pretrained using large collections of unlabeled images. Their approach follows a SimCLR-style paradigm that learns representations by comparing multiple augmented views of the same image. Importantly, the study reported performance levels comparable to those achieved with ImageNet pretraining (approximately +5–7pp improvement), while enabling the model to learn features more closely aligned with anatomical structures rather than patterns derived from unrelated natural image domains. This strategy also offers substantial scalability, since hospital archives typically contain millions of unlabeled CXRs that can be used for representation learning. As a result, it challenges the traditional dependence on ImageNet-based initialization. Nevertheless, a comprehensive comparison between different initialization strategies—including supervised ImageNet pretraining, supervised domain-specific pretraining on large labeled CXR datasets such as ChestX-ray14 and CheXpert, and self-supervised contrastive pretraining—remains limited. Determining which of these strategies provides the most effective initialization therefore remains an open research question.

To further mitigate overfitting in data-constrained settings, several studies have investigated advanced regularization techniques. For example, the HyCoViT framework incorporates the MixUp augmentation strategy, which combines pairs of images and their corresponding labels using randomly sampled mixing coefficients to generate synthetic training examples (Naghash et al., 2025). This process encourages the model to learn smoother decision boundaries and improves generalization. However, such interpolation-based augmentation may also introduce certain limitations. In particular, blending images can reduce the clarity of subtle lesion boundaries, which may negatively affect the localization of small abnormalities such as nodules or early-stage infiltrates. Dynamic Dropout algorithms balance exploration capacity in early training with memorization prevention in later epochs by adapting regularization strength across training phases. In contrast, classical preprocessing approaches including anisotropic diffusion filtering and contrast-limited histogram equalization (Ayalew et al., 2024) provide minimal benefit in modern contexts where convolutional layers automatically learn optimal preprocessing through batch normalization.

### 2.3 Explainability, clinical validation, and deployment

Clinical deployment requires interpretability mechanisms that enable physicians to verify model reasoning, identify failure modes, and establish trust in automated diagnostic outputs. Gradient-weighted Class Activation Mapping can be used to create coarse localizations as heatmaps, whereas Attention-based Visualizations (Guan et al., 2020; Ouyang et al., 2021) may have a direct relationship with an attention mechanism allowing for native explanations of the data without having to perform *post-hoc* processing on it. Diwakar et al., proposed a method of PCA-based Localization using Principal Component Analysis (PCA) of feature maps to obtain a Dice Similarity Coefficient of 97.5%. Despite this progress, a substantial gap remains: very few studies have evaluated the clinical usefulness of these methods through radiologist-agreement experiments that quantitatively assess whether model-generated explanations align with radiologists' areas of focus, bolster their diagnostic confidence, or assist in detecting errors. As a result, no standardized clinical evaluation framework for explainability currently exists.

Real world clinical validation is broader than the review of a retrospectively collected dataset; therefore, real world clinical validation requires the evaluation of the technology being used in an operational environment. Ye et al. (2024) have demonstrated that ARDS can be effectively surveilled using CXRs from 15,899 ICU patients, achieving sensitivities of 91.8%–97.8% and specificities of 96.6%–8.8%. The authors demonstrated that automated CXR analysis yields clinically useful results (i.e., < 1 min) compared to electronic health record surveillance (i.e., 12–24 h), and their two-stage approach to analysis (i.e., Normal vs. Abnormal screening as the first stage, followed by disease specific classification as the second stage) matches clinical workflow for rapid triage. Clinical environments vary significantly in terms of how they tradeoff between sensitivity and specificity; in ICU settings where the clinical consequence of a false negative is catastrophic, sensitivity is given higher priority than specificity, and conversely, in outpatient screening settings, specificity is given higher priority to avoid unnecessary downstream testing. The higher sensitivity achieved by Ye et al. (2024) in ICU settings is consistent with these clinical setting based priorities, however it also increases the false positive rate which has been shown to contribute to alarm fatigue and remains an open issue in the development of clinical decision support systems.

Most contemporary studies evaluate models retrospectively using curated datasets with relatively controlled image quality, which limits the assessment of real-world generalizability. Reported performance is strongly influenced by dataset characteristics; data collected from a single institution often fail to reflect variations in imaging equipment, acquisition protocols, and patient populations across different clinical settings. Labeling practices introduce further complications, as disease definitions may vary between radiologists and institutions, and many datasets derive ground truth from radiology reports rather than confirmed diagnoses. In addition, subtle abnormalities often show low inter-rater agreement even among experts. These issues make direct comparison of model performance across studies difficult when different datasets and curation standards are used.

### 2.4 Performance claims and methodological rigor

Recent literature shows evidence of performance variability, with accuracy rates oscillating from 81% to over 99%, which raises concerns regarding the legitimacy of such findings. In cases of research with almost perfect accuracy (≥99%), it is evident that such research is plagued with concerns such as failing to report variance, using only one split of data for cross-validation, weak baselines, and publication in venues with weak review. In such cases, the sheer number of 99%-plus claims may indicate leaking, overfitting, or testing on too simple data sets, rather than actual advancements in methodology. A systematic review of 68 CXR classification studies done by Meedeniya et al. (2022). supports these claims, which indicate that although 61.8% of the studies used transfer learning, the accuracy of both transfer learning techniques (92.2%) and models trained from scratch (92.5%) is almost similar. This indicates that it is not the training methodology but the testing methodology.

Models that have been evaluated with more transparent methodologies often appear more reliable, even if they have marginally worse performance. For example, Mirugwe et al. (2025) were able to achieve 99.4% accuracy in binary tuberculosis detection using a model based on VGG16. This is because binary detection is made comparatively simpler by unique radiographic features of tuberculosis, which include cavitation and nodules. However, HyCoViT claimed that it had been 98.81% accurate in multi-class pneumonia detection (Naghash et al., 2025). This is because features of pneumonia, which include infiltrates, consolidations, and opacities, make it difficult to differentiate them. Such comparisons illustrate the need for context in assessing model performance rather than relying on raw accuracy figures.

There is also still a lot of discussion about which evaluation metrics best show how useful something is in a clinical setting. It's easy to understand accuracy, but it can be misleading in class-imbalanced datasets where predictions for the majority class get scores that are too high. Sensitivity and specificity are more clinically relevant but depend on the choice of threshold, which affects the balance between false positives and false negatives. The Area Under the Receiver Operating Characteristic Curve (AUC) provides threshold-independent assessment; however, it may obscure performance at clinically significant operating points. As a result, there is still no agreement on which metric is the best way to predict how it will work in the real world. Sometimes, the choice of metric can also affect how results are shown.

### 2.5 Open challenges and motivation for HyRA-CXR

Current research on CXR classification continues to encounter numerous unresolved challenges that hinder clinical application. A significant inquiry pertains to the most effective pretraining methodologies. Many people use ImageNet pretraining because it is easy, but its features come from natural images instead of radiographic data. Pretraining that is specific to a certain field may give you more useful representations, but it costs a lot of money to annotate. So, even though Gazda et al. (2021) shows that self-supervised methods can get good results, many studies still use ImageNet initialization by default.

There is also a lot of variation in methodological rigor. Reports of near-perfect accuracy (≥99%) frequently accompany limited variance reporting, dependence on single train-test splits, inadequate baseline comparisons, or assessments conducted on simplified datasets. Furthermore, the robustness across deployment environments is still not well understood. Numerous models are assessed using single-institution datasets, offering limited understanding of performance amidst variations in equipment, acquisition protocols, or patient demographics.

Explainability techniques are frequently suggested but seldom subjected to clinical validation. There are no standard ways in the field to measure explainability through radiologist agreement or other ways to see how well it fits with expert diagnostic reasoning.

It is also rare to do prospective clinical validation. Transitioning from research prototypes to practical implementation generally necessitates external validation, prospective studies, and regulatory approval, resulting in a disparity between algorithmic development and clinical application. Meedeniya et al. (2022) also point out that there aren't enough datasets, that there are too many classes, and that there isn't a standardized way to evaluate explainability. They stress the need for clear evaluation methods and evidence-based model selection instead of just new architecture.

On a larger scale, the field needs to strike a balance between quickly developing new architectures and using them in practical clinical settings. Improvements in benchmark performance have been driven by CNN-based models, attention mechanisms, and hybrid models. However, with regards to datasets, there is still difficulty in dealing with small sample sizes, inconsistent labeling, and bias toward a particular institution. To make an impact in clinical settings, we not only need better accuracy but also faster computation, explanation, reliability across situations, and open evaluation.

These issues are what inspired the development of the HyRA-CXR. The suggested model has various architectural features that seek to address the issues that were previously experienced. The model was developed from scratch with hyperparameter optimization carried out in the process. The architectural features of the HyRA-CXR comprise the residual blocks and the double attention. The skip connections were incorporated to facilitate the smooth flow of gradients and the adaptive refinement of features. The approach ensures that the features are maintained in the attention-based approach without the resource-intensive features of the Vision Transformer.

HyRA-CXR employs five-fold stratified cross-validation with full variance reporting instead of single-split evaluation to make the methods more rigorous. The performance of the model was analyzed in a number of ways, such as precision, recall, F1 scores, confusion matrices, and learning curves. This gives us an overall understanding of how well the model is performing. Ablation experiments used to get a deeper insight into the role of architectural components of the model. The model does not only concentrate on increasing the accuracy of the model but also on balancing sensitivity and specificity for various classes of diagnosis, as clinical scenarios can be different in various cases.

## 3 Proposed HyRA-CXR model

**Figure 1:**
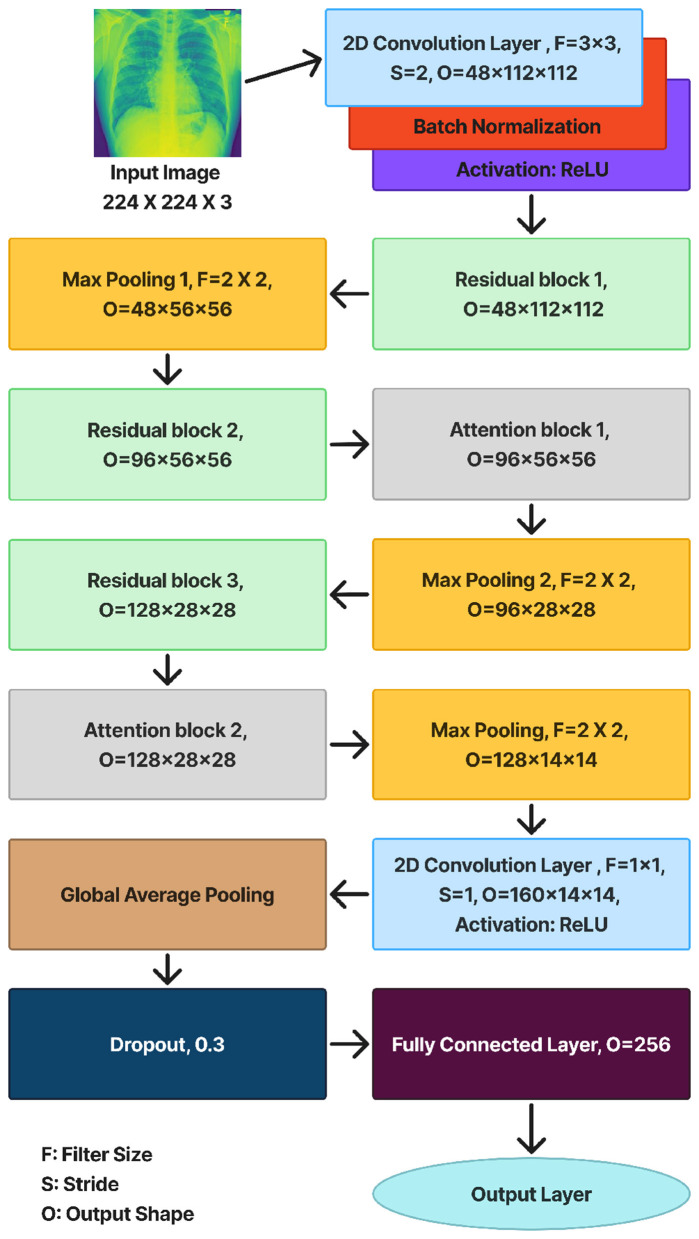
HyRA-CXR model architecture.

The proposed HyRA-CXR model is formulated as a hybrid deep convolutional neural network (CNN) for chest X-ray image classification. The architecture combines lightweight residual blocks with channel-attention mechanisms to improve feature representation while preserving computational efficiency. Figure 1 presents the overall structure of the network, showing the progression from the input image through the feature extraction stages to the final classification layer.

An input image of size *224* × *224* × *3* is first processed by an initial convolutional layer with a kernel size of *3* × *3* and a stride of 2, serving as the primary feature extractor. This layer is followed by batch normalization (BN) to stabilize the learning process and a ReLU activation function to introduce non-linearity and accelerate convergence. Then we have three Residual Blocks, as illustrated in Figure 1. Each Residual Block consists of two *3* × *3* convolutional layers, each followed by batch normalization (BN) and a ReLU activation. The output of these convolutional operations is then added to the original input through a shortcut connection, which helps the network preserve important spatial information and maintain stable gradient flow across the network. This shortcut connection can be either:

an *identity shortcut*, used when the input and output feature dimensions are the same; or

a *projection shortcut* (implemented using a *1* × *1* convolution), applied when the dimensions differ. Finally, a ReLU activation is applied to the summed output to generate the final feature map of the Residual Block.

After the second and third Residual Blocks, an Attention Block is applied to refine the extracted features. This block integrates both *channel* and *spatial* attention mechanisms to emphasize diagnostically relevant regions. The *channel attention module* emphasizes the most informative feature channels by applying global average pooling followed by two fully connected layers—one for dimensionality reduction and another for channel-wise recalibration using a sigmoid activation. While the *spatial attention module* highlights the most significant spatial regions within the feature map by computing an average projection across channels, convolving it with a *7* × *7* kernel, and applying a sigmoid activation to produce a spatial attention mask. The final attended feature map is obtained by element-wise multiplication of the input features with both the channel and spatial attention maps, ensuring that the network concentrates on critical lung areas while suppressing irrelevant background information.

The final part of the HyRA-CXR architecture is the Head Layer, which transforms the high-level feature maps into class probabilities. A *1* × *1* convolution is first applied to adjust the feature dimensionality, followed by a Global Average Pooling layer to aggregate spatial information into a compact feature vector. The resulting vector is passed through a fully connected layer of 256 neurons with a ReLU activation to enhance non-linear feature interaction and improve class separability. Finally, a Softmax layer outputs the probability distribution across the three diagnostic categories: *Normal, Lung Opacity*, and *Viral Pneumonia*.

## 4 Experiments and evaluation

This section presents the experimental results of the proposed HyRA-CXR model. The experiments were designed to evaluate the effectiveness of the model in classifying chest X-ray images into three diagnostic categories: *Normal, Lung Opacity*, and *Viral Pneumonia*. The experimental procedure covers dataset description, preprocessing stages, hyperparameter optimisation, and performance evaluation using a stratified k-fold cross-validation framework.

### 4.1 Dataset

The proposed HyRA-CXR model was trained and evaluated using the publicly available Lung X-Ray Image Dataset hosted on Mendeley Data Contributor by Talukder (2023). This dataset comprises a total of 3,475 chest X-ray images categorized into three diagnostic classes: *Normal* (1,250 images), *Lung Opacity* (1,125 images), and *Viral Pneumonia* (1,100 images).

All chest X-ray images were preprocessed before being fed into the proposed HyRA-CXR model. Each image in the dataset was resized to 224 × 224 to match the model's input dimension and normalized to the range [0,1]. Data augmentation was applied to the training set to improve generalization and mitigate overfitting, incorporating random horizontal flips, small rotations, translations, zoom operations, and contrast adjustments.

### 4.2 Experimental setup

All experiments were conducted on a personal computer with an Intel(R) Core(TM) i7-10750H CPU (Intel Corporation, Santa Clara, CA, USA) @ 2.60 GHz, 16 GB RAM, and an NVIDIA GeForce GTX 1660 Ti GPU (6 GB VRAM), running on Windows 10. The implementation was developed in Python 3.9.7 (Python Software Foundation, Wilmington, DE, USA) using the TensorFlow 2.17.1 and Keras 3.10.0 frameworks.

### 4.3 Hyperparameter

To determine the optimal configuration of the proposed HyRA-CXR model, a hyperparameter optimization was performed using the KerasTuner framework with a Random Search strategy over 30 independent trials. Each trial was trained for up to 100 epochs using a batch size of 16. The dataset was randomly split into 80% for training and 20% for validation. The trial that achieved the highest validation accuracy was selected as the optimal set of hyperparameters.

The search space included the *L*_2_ regularization weight (λ), dropout rate (*p*), number of filters for each residual block (Filt-RB1, Filt-RB2, Filt-RB3), head filters (Filt-Head), optimizer type, and learning rate (LR). In our model design, Filt-RB1 is applied to both the initial convolutional layer and Residual Block 1; Filt-RB2 is shared between Residual Block 2 and the first Attention Block; and Filt-RB3 is used for Residual Block 3 and the second Attention Block. The best configuration achieved a validation accuracy of 93.5%. Table 1 summarizes the explored hyperparameter search space and the selected optimal values, while Table 2 provides a detailed summary of all 30 tuning trials.

**Table 1:** Hyperparameter search space and optimal configuration for the HyRA-CXR model.

Hyperparameter (symbol)	Search space	Best value
*L*_2_ regularization weight (λ)	{1 × 10^−4^, 5 × 10^−5^, 1 × 10^−5^}	5 × 10^−5^
Dropout rate (*p*)	[0.2, 0.6] (step 0.1)	0.3
Filters RB1/Initial Conv	{24, 32, 48}	48
Filters RB2 + Attention 1	{48, 64, 96}	96
Filters RB3 + Attention 2	{96, 128, 160}	128
Head filters (Filt-Head)	{160, 192, 256}	160
Optimizer	{Adam, AdamW, RMSProp}	RMSProp
Learning rate (LR)	{1 × 10^−3^, 5 × 10^−4^, 3 × 10^−4^, 1 × 10^−4^}	1 × 10^−3^
Best validation accuracy	–	93.5%

**Table 2:** Summary of the 30 KerasTuner trials for the HyRA-CXR model.

Trial ID	Val. Acc.	Val. Loss	λ	*p*	Filt-RB1	Filt-RB2	Filt-RB3	Filt-Head	Optimizer	LR
1	0.927	0.334	1e-5	0.20	32	64	96	192	RMSProp	0.001
2	0.924	0.379	5e-5	0.30	32	96	96	160	RMSProp	0.001
3	0.917	0.227	1e-5	0.30	32	64	160	192	AdamW	0.0001
4	0.925	0.408	1e-4	0.20	48	96	96	192	Adam	0.0003
5	0.922	0.416	1e-5	0.40	32	64	96	160	AdamW	0.0001
6	0.928	0.355	1e-4	0.40	48	48	96	160	AdamW	0.001
7	0.928	0.338	1e-4	0.20	32	64	160	256	AdamW	0.0005
8	0.931	0.347	5e-5	0.40	24	96	96	256	Adam	0.0003
9	0.931	0.347	1e-5	0.30	24	64	128	160	AdamW	0.001
10	0.927	0.348	1e-4	0.60	48	48	160	192	RMSProp	0.001
11	0.925	0.354	1e-5	0.20	32	48	160	160	Adam	0.0003
12	0.919	0.279	1e-5	0.30	32	64	128	256	RMSProp	0.001
13	0.924	0.442	1e-4	0.20	24	96	160	160	AdamW	0.0001
14	0.915	0.367	1e-4	0.40	32	48	128	256	AdamW	0.0001
15	0.927	0.375	1e-4	0.40	32	96	160	192	Adam	0.0005
16	0.921	0.504	1e-4	0.40	24	48	96	160	RMSProp	0.0003
17	0.921	0.390	5e-5	0.50	32	64	96	192	Adam	0.0005
18	0.921	0.442	1e-4	0.60	24	96	128	160	AdamW	0.001
19	0.911	0.401	5e-5	0.30	48	64	96	256	AdamW	0.0001
20	0.927	0.337	1e-5	0.40	48	96	96	192	Adam	0.001
21	0.924	0.268	5e-5	0.20	32	64	96	256	AdamW	0.001
22	0.929	0.378	5e-5	0.40	32	48	160	160	Adam	0.0003
23	0.922	0.425	5e-5	0.50	48	48	96	192	Adam	0.0005
24	0.931	0.500	5e-5	0.40	48	96	160	160	AdamW	0.0005
**25**	**0.935**	**0.481**	**5e-5**	**0.30**	**48**	**96**	**128**	**160**	**RMSProp**	**0.001**
26	0.918	0.547	5e-5	0.60	48	48	96	192	AdamW	0.0003
27	0.928	0.459	5e-5	0.40	48	96	128	160	AdamW	0.0003
28	0.906	0.349	1e-5	0.20	24	64	128	256	RMSProp	0.0001
29	0.934	0.483	1e-4	0.30	48	48	96	192	AdamW	0.0005
30	0.915	0.309	1e-4	0.50	32	48	128	192	AdamW	0.001

The tuning results (Table 2) show that Trial #25 achieved the highest validation accuracy of 93.5% using λ = 5 × 10^−5^, dropout *p* = 0.3, and filter configuration (48–96–128–160) with the RMSProp optimizer and a learning rate of 0.001. This configuration provided a balanced compromise between regularization and training stability. The small *L*_2_ penalty together with moderate dropout helped reduce overfitting while preserving useful feature representations. In addition, the gradual increase in filter numbers allowed hierarchical feature extraction across residual and attention blocks, improving the model's ability to focus on abnormal regions.

The hyperparameter search space was defined to balance architectural flexibility with training stability. The chosen ranges for regularization, dropout, learning rate, and filter sizes allow exploration of model capacity while avoiding extreme configurations that could destabilize optimization.

Hyperparameter tuning was conducted using an 80%/20% development split to determine a single configuration. After selection, these hyperparameters were fixed and the final performance was estimated using stratified five-fold cross-validation. Although this procedure is not fully nested and may introduce a slight optimistic bias, it improves computational efficiency.

### 4.4 Evaluation metrics

The performance of the HyRA-CXR model was assessed using four commonly used metrics: Accuracy, Precision, Recall, and F1-score. Accuracy represents the proportion of correctly classified samples across the dataset, while Precision and Recall indicate the model's ability to limit false positives and false negatives, respectively. The F1-score, defined as the harmonic mean of Precision and Recall, provides a balanced measure of classification performance. These metrics offer complementary perspectives on the model's predictive behavior and are widely applied in image classification studies (Farhan et al., 2025b,a). The evaluation metrics are defined as in Equations 1–4.


$$\begin{array}{lll} \text{Accuracy} & = & \frac{TP+TN}{TP+TN+FP+FN} \end{array}$$ (1)

$$\begin{array}{lll} \text{Precision} & = & \frac{TP}{TP+FP} \end{array}$$ (2)

$$\begin{array}{lll} \text{Recall} & = & \frac{TP}{TP+FN} \end{array}$$ (3)

$$\begin{array}{lll} \text{F1-score} & = & 2\times \frac{\text{Precision}\times \text{Recall}}{\text{Precision}+\text{Recall}} \end{array}$$ (4)

where *TP*, *TN*, *FP*, and *FN* denote the numbers of true positives, true negatives, false positives, and false negatives, respectively.

### 4.5 Results

To evaluate the generalization performance of the proposed HyRA-CXR architecture, a stratified five-fold cross-validation was performed. Each fold utilized 80% of the dataset for training and 20% for validation, ensuring a balanced representation of all diagnostic classes. The BATCH_SIZE was set to 16, and each trial was trained for up to 100 epochs with EarlyStopping and ReduceLROnPlateau callbacks to prevent overfitting. Each fold (F1–F5) represents an independent training–validation cycle, ensuring that every sample in the dataset was used once for validation. Table 3 presents the complete performance summary for all five folds. The model showed stable performance across folds, achieving a mean validation accuracy of 90.39% with a standard deviation of 1.30 pp. Similarly, the mean precision, recall, and F1-score reached 90.66%, 90.64%, and 90.57%, respectively, each with a standard deviation below 1.3 pp, confirming the robustness of the proposed model.

**Table 3:** The summary of the five-fold cross-validation results.

Metric (%)	F1	F2	F3	F4	F5	Mean ± Std
Accuracy	89.93	90.22	88.63	90.50	92.66	**90.39 ± 1.30**
Precision	90.20	90.47	88.97	90.78	92.89	**90.66 ± 1.27**
Recall	90.23	90.32	89.00	90.90	92.76	**90.64 ± 1.23**
F1-score	90.14	90.37	88.86	90.65	92.82	**90.57 ± 1.28**

Bold values indicate the best or most significant results among the compared methods.

Across all folds, the model maintained validation accuracies ranging from 88.6% to 92.7%, with consistent convergence and no significant overfitting. In the first and second folds, both training and validation accuracy plateaued around 90%, while subsequent folds (F4 and F5) achieved slightly higher values. Also, The averaged results across all folds yield a mean validation accuracy of 90.39% and a standard deviation of 1.30 percentage points, reflecting the stability of the optimized hyperparameters obtained from the KerasTuner search.

**Figure 2:**
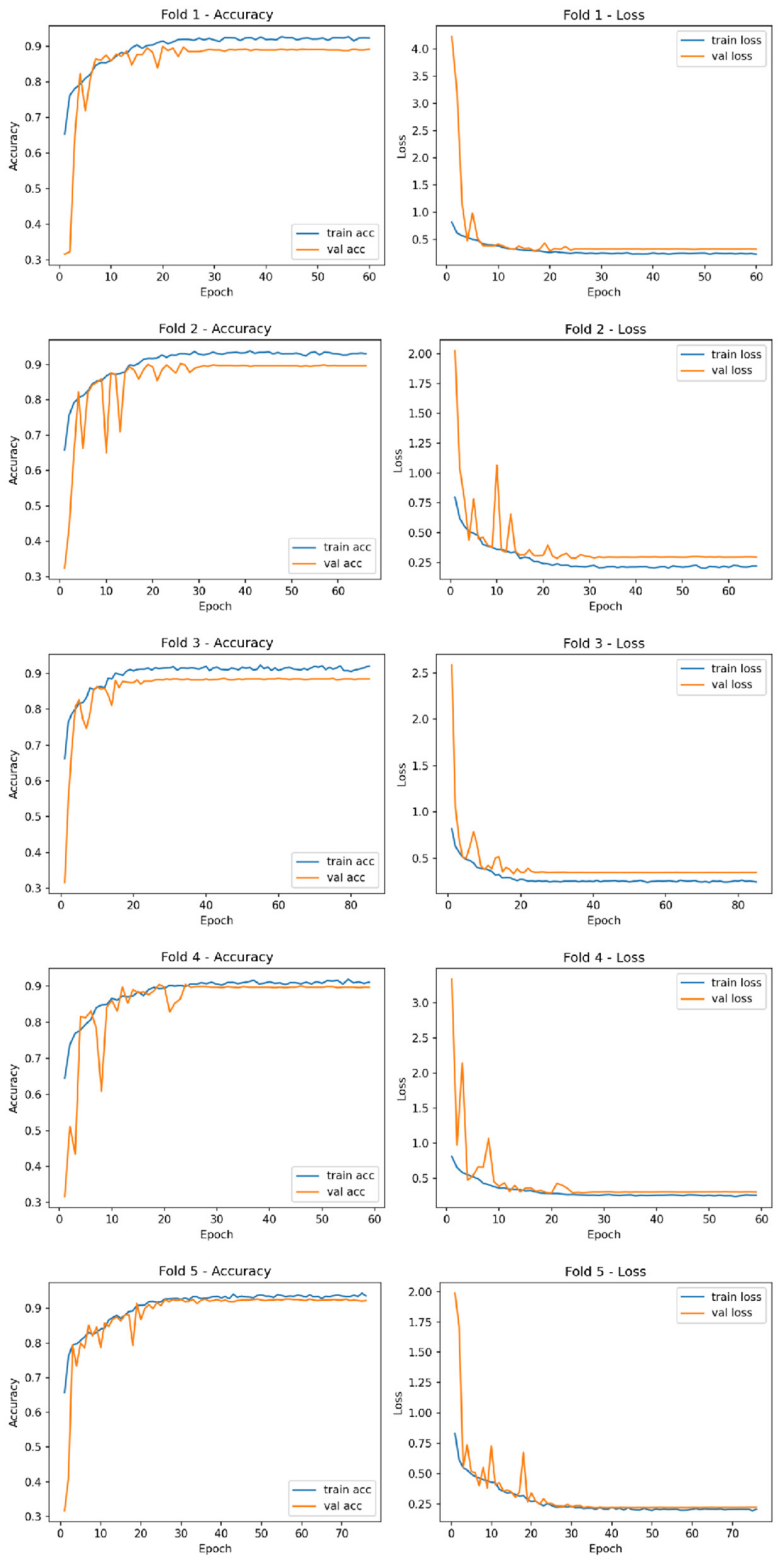
Training and validation accuracy/loss curves for the five folds.

The learning curves in Figure 2 show that the training and validation losses decreased sharply during the initial epochs and stabilized after approximately 30–40 epochs, indicating efficient and stable learning dynamics. Similarly, the validation accuracy curves demonstrate minimal divergence from the training curves, confirming the model's resilience against overfitting.

**Figure 3:**
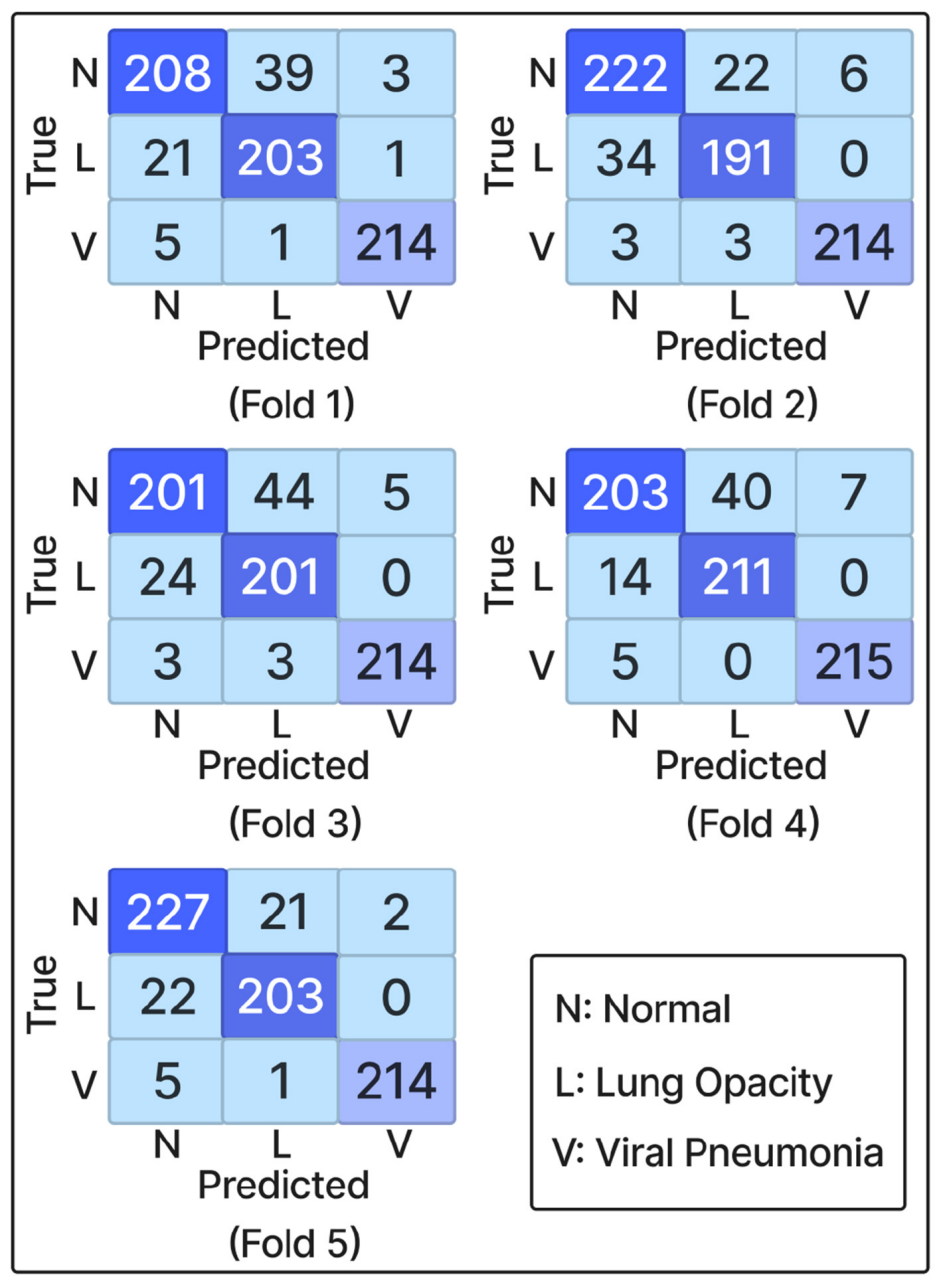
Confusion matrices across the five folds.

Analysis of the confusion matrices in Figure 3 reveals that the *Viral Pneumonia* class achieved the most consistent classification performance across all folds, with minimal false positives or negatives. The *Normal* and *Lung Opacity* classes showed moderate overlap due to visual similarities in mild opacity cases.

### 4.6 External dataset evaluation (generalizability)

To further assess the generalizability of HyRA-CXR beyond the primary dataset, we conducted an external evaluation using the publicly available *Chest X-Ray Images (Pneumonia)* dataset (Mooney, 2018; Kermany et al., 2018). This dataset contains two classes (NORMAL and PNEUMONIA) and provides predefined train, validation, and test splits.

The dataset distribution is summarized as follows: 5,216 training images (1,341 NORMAL and 3,875 PNEUMONIA), 16 validation images (8 per class), and 624 test images (234 NORMAL and 390 PNEUMONIA). All images were resized to 224 × 224 and normalized to [0, 1]. The same augmentation strategy and training configuration used in the primary experiments were applied to ensure methodological consistency.

**Figure 4:**
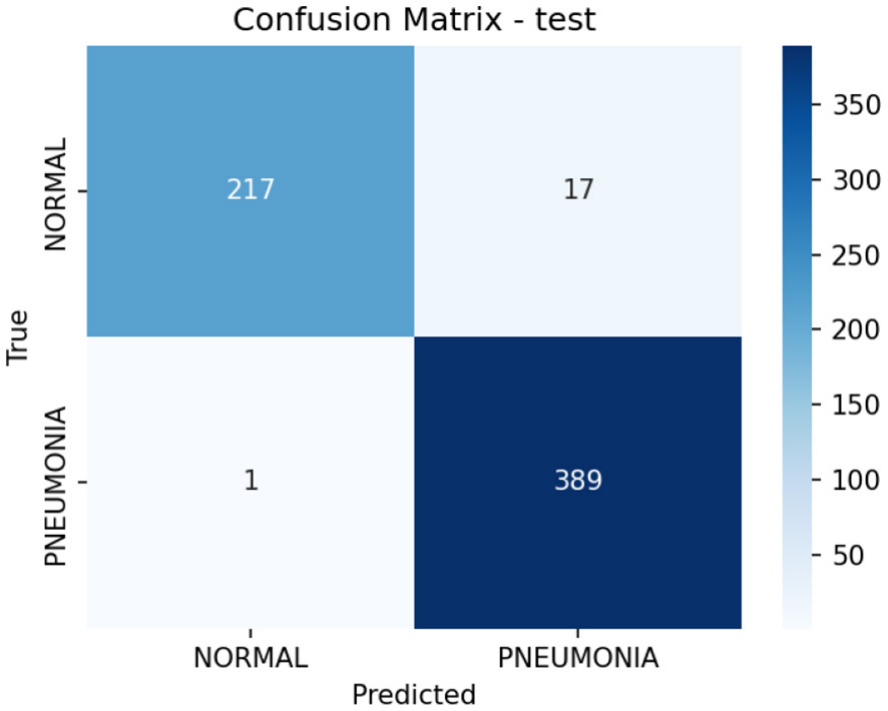
Confusion matrix on the Kaggle dataset.

As shown in Table 4, HyRA-CXR achieved a test accuracy of 97.12% with a F1 score of 96.88% on the test set. These results indicate transferability to an independent dataset. Also, the confusion matrix for the test split is presented in Figure 4. Only 17 NORMAL cases were misclassified as PNEUMONIA and 1 PNEUMONIA case was misclassified as NORMAL, indicating recognition capability across both categories.

**Table 4:** External evaluation on the Kaggle Chest X-Ray (pneumonia) dataset.

Accuracy (%)	Precision (%)	Recall (%)	F1 (%)
97.12	97.68	96.24	96.88

Overall, these results show that HyRA-CXR transfers well to an independent dataset under a related binary classification setting. However, this evaluation differs from the primary three-class task; therefore, it should be interpreted as complementary evidence rather than a direct measure of three-class cross-dataset generalization.

We note that the provided validation split in this dataset is relatively small; hence, the reported results focus on test-set performance.

### 4.7 Compare the results of the HyRA-CXR model with other models

To evaluate the proposed HyRA-CXR model, three baselines (DenseNet121, Xception, and MobileNetV3-Large) were trained and evaluated under identical experimental conditions. All models were trained using five-fold stratified cross-validation with an 80%-20% train–validation split per fold, an input resolution of 224 × 224, and a batch size of 16. Each model adopted a unified classification top layers composed of GlobalAveragePooling, Dropout (0.3), Dense (256, ReLU), and Softmax (3) layers.

Table 5 presents the fold-wise validation accuracy summaries for all models.

**Table 5:** Comparison the validation accuracy between HyRA-CXR and the other CNN models.

Model	F1	F2	F3	F4	F5	Mean (%)
**HyRA-CXR**	89.93	90.22	88.63	90.50	92.66	**90.39**
DenseNet121	89.50	89.06	88.49	90.79	91.08	89.38
Xception	89.06	88.92	88.78	90.79	88.06	89.12
MobileNetV3-Large	64.75	60.29	63.60	67.05	61.44	63.83

Bold values indicate the best or most significant results among the compared methods.

The proposed HyRA-CXR model achieved the highest overall average accuracy of 90.39%, surpassing both DenseNet121 89.38% and Xception 89.12%. In contrast, MobileNetV3-Large shows lower performance 63.83%, indicating its limited capacity for complex feature extraction in medical imaging tasks.

To assess the statistical significance of the observed performance differences, paired *t*-tests were conducted across the five cross-validation folds comparing HyRA-CXR against each baseline model. The comparison with DenseNet121 yielded (*t* = 1.78, *p* = 0.15), and with Xception (*t* = 1.43, *p* = 0.23), indicating that the observed improvements over these strong CNN baselines, although numerically higher, are not statistically significant at the 0.05 level. However, the performance difference compared to MobileNetV3-Large was statistically significant (*t* = 17.74, *p* < 0.001), confirming the superior stability and discriminative capability of HyRA-CXR over lightweight architectures.

Table 6 highlights the compact nature of HyRA-CXR. With only 0.52 million parameters, the proposed architecture is significantly smaller than DenseNet121 (7.30M) and Xception (21.38M), while still achieving the highest classification accuracy among the evaluated models. Although MobileNetV3-Large exhibits moderate compactness (3.24M parameters), its accuracy was substantially lower under identical evaluation conditions. These findings demonstrate that HyRA-CXR achieves a favorable balance between model compactness and diagnostic performance.

**Table 6:** Models complexity comparison.

Model	Total parameters	Accuracy (%)
HyRA-CXR	0.52M	90.39
DenseNet121	7.30M	89.38
MobileNetV3-Large	3.24M	63.83
Xception	21.38M	89.12

### 4.8 Ablation study

To further analyze the contribution of architectural modules within HyRA-CXR, an ablation study was performed. Two modified variants of the model were trained and evaluated under the same five-fold cross-validation setup and hyperparameters: (1) a variant without attention modules, and (2) a variant without residual connections. All experiments used identical preprocessing, and training configurations.

The Table 7 show removing either component results in a decline in classification performance, confirming that both residual and attention modules contribute meaningfully to the proposed model performance. Eliminating the residual blocks slightly degraded stability and overall accuracy (89.67%), while omitting attention modules led to a comparable drop (89.76%), indicating their complementary effects. The complete HyRA-CXR model achieved the highest average accuracy (90.39%), validating the benefit of jointly employing residual and attention mechanisms for enhanced model performance.

**Table 7:** Comparison accuracy between the full HyRA-CXR model and its ablated variants.

Model variant	F1	F2	F3	F4	F5	Mean (%)
HyRA-CXR (full)	89.93	90.22	88.63	90.50	92.66	**90.39**
Without attention	89.91	89.73	89.58	90.01	89.50	**89.76**
Without residual	89.88	89.34	89.22	90.11	89.07	**89.67**

Bold values indicate the best or most significant results among the compared methods.

### 4.9 Explainability analysis (XAI)

To understand how the proposed HyRA-CXR model makes the prediction decision, explainable AI (XAI) techniques were integrated to visualize the regions that contributed most to each prediction. We chose two methods for the interpretability: (1) Gradient-weighted class activation mapping (Grad-CAM) is a method that highlights discriminative regions influencing the predicted class (Selvaraju et al., 2017; Farhan et al., 2025c). (2) Spatial attention maps, which are generated natively from the attention modules within HyRA-CXR, revealing feature importance learned during training. The goal was to inspect whether the model's highlighted regions are anatomically plausible (e.g., concentrated within lung fields) and whether the model appears to rely on obvious artifacts.

**Figure 5:**
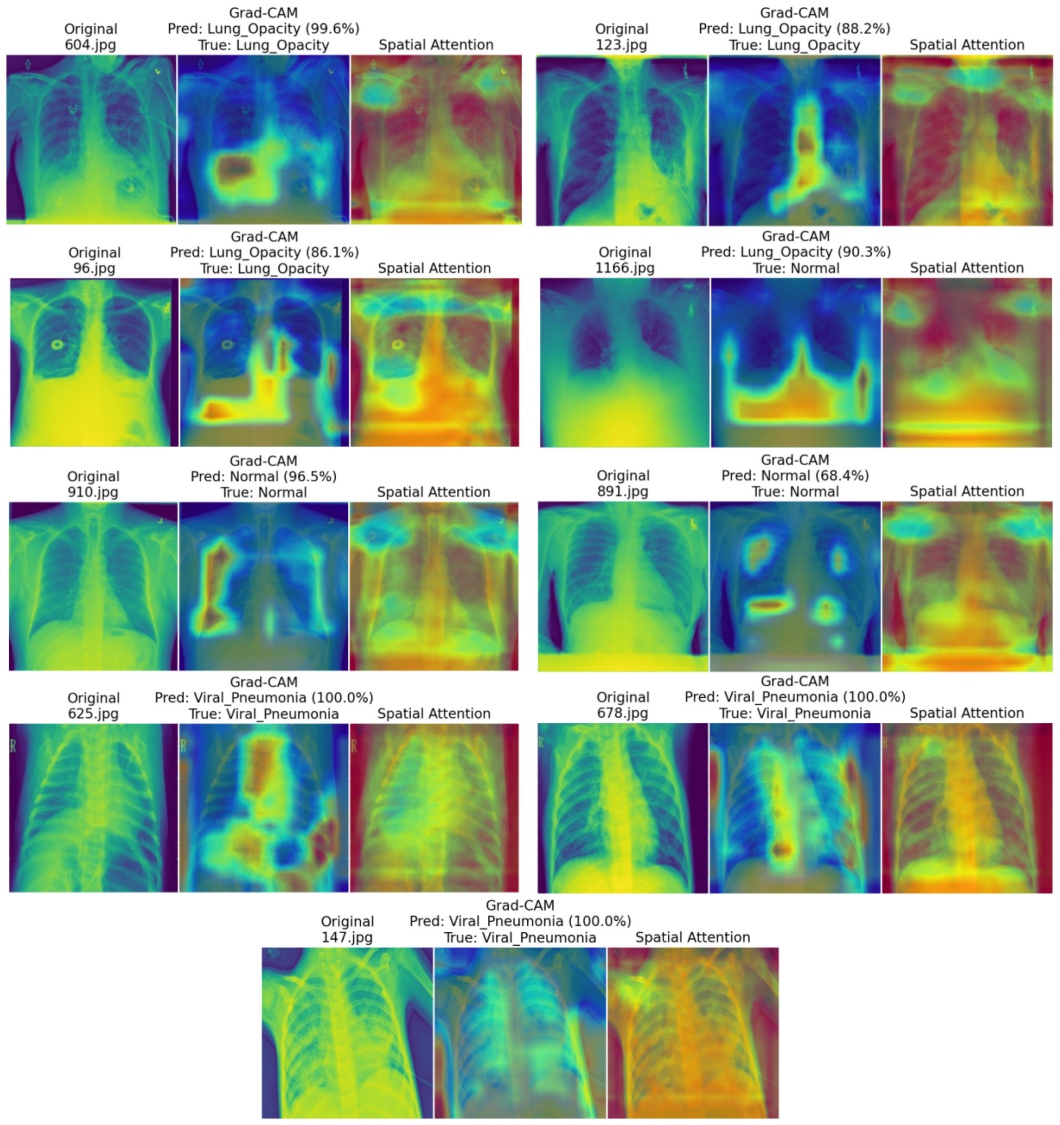
Grad-CAM and spatial attention visualization maps for the HyRA-CXR model.

A representative set of nine test images (three per class) was analyzed. The Figure 5 shows the combined visualization output produced using the proposed HyRA-CXR. Grad-CAM highlighted the most meaningful radiological lung regions contributing to each prediction rather than artifacts, borders, or textual labels. spatial attention maps further demonstrated that the network's attention blocks prioritized anatomical structures associated with each disease class. The combined XAI outputs provided deeper insight into both correct and incorrect predictions. Overall, these visualizations improve transparency by enabling qualitative inspection of model behavior and can support future clinician-in-the-loop validation prior to deployment.

## 5 Discussion

Achievement of Research Questions. The experimental results provide clear answers to the three research questions posed in this study. Regarding **RQ1**, the ablation study (Table 7) confirms that residual and attention modules contribute synergistically. Removing the residual blocks reduced accuracy from 90.39% to 89.67% (−0.72 pp), while removing the attention modules reduced accuracy to 89.76% (−0.63 pp). The fact that removing either component independently degrades performance demonstrates that neither subsumes the other; rather, residual connections stabilize gradient flow enabling deeper feature extraction, while attention mechanisms refine these features by emphasizing diagnostically relevant regions. Regarding **RQ2**, the five-fold stratified cross-validation results (Table 3) demonstrate that HyRA-CXR achieves a mean accuracy of 90.39% ± 1.30%, outperforming DenseNet121 (89.38%) and Xception (89.12%) under identical experimental conditions (Table 5). This was achieved with only 0.52M parameters and systematic KerasTuner optimization across 30 configurations, confirming that a lightweight hybrid architecture can achieve competitive performance under rigorous evaluation. Regarding **RQ3**, Grad-CAM and native attention visualizations (Figure 5) provide *qualitative* evidence that HyRA-CXR primarily attends to the lung fields in many cases, rather than relying on obvious spurious cues such as image borders or textual markings. We emphasize that this is not a clinical validation of localization: due to the lack of pixel-level annotations and expert review, these visualizations should be interpreted as transparency tools that help inspect model behavior and motivate future quantitative and clinician-in-the-loop evaluation.

The results obtained show the effectiveness of the proposed HyRA-CXR model in achieving stable classification of chest X-ray images. The integration of residual and attention mechanisms meaningfully contributed to feature representation and generalization, as evidenced by the ablation study results.

The proposed model, when compared with DenseNet121, Xception, and MobileNetV3-Large, demonstrates higher mean accuracy. This improvement can be attributed to the complementary nature of residual and attention pathways, where residual connections facilitate efficient gradient propagation and deeper learning, while the attention blocks refine feature localization by emphasizing diagnostically relevant regions. The ablation study further supported these findings. Excluding either the residual blocks or the attention modules resulted in a noticeable drop in accuracy (89.67% and 89.76%, respectively), highlighting their role in enhancing the model's discriminative ability. In addition, the learning curves showed more stable validation accuracy and smoother loss behavior for the complete HyRA-CXR architecture, indicating improved robustness to overfitting and greater consistency across folds.

It is important to note that this study uses a three-class single-label classification setting. In the real world, however, chest X-ray interpretation often involves multi-label diagnosis with several coexisting pathologies. The selected formulation provides a regulated setting for examining architectural design and feature learning within distinctly defined categories. This setup makes it easier to do systematic evaluations and comparisons, but in the future, it will need to be expanded to include multi-label classification so that it better matches clinical practice.

Inspection of the confusion matrices indicated that the model maintained strong sensitivity and specificity across all classes. The most frequent misclassifications occurred between *Lung Opacity* and *Viral Pneumonia*, likely due to their overlapping radiographic characteristics. Overall, HyRA-CXR demonstrates that an effective integration of architectural components can improve CXR classification performance.

The XAI analysis also showed that HyRA-CXR makes visual explanations for its predictions that are anatomically meaningful. The attention maps consistently emphasized clinically significant areas in normal, opacity, and viral pneumonia images. This level of openness makes it easier to understand and suggests that it could be used as a decision-support system, but more clinical testing is needed.

## 6 Limitations and future directions

This study has several limitations; for example, the three-class formulation used here oversimplifies clinical reality, where radiologists often look at more than one condition at a time. To make the framework more realistic, it should be changed to multi-label classification. Also, while training from scratch sets a clear baseline, it might not be able to show how well performance can be improved. Previous studies have shown that pretraining strategies can improve performance by 4–7 percentage points (Gazda et al., 2021).

Future research ought to rectify these limitations via multi-dataset validation, expansion to multi-label learning, and a systematic comparison of pretraining methodologies (ImageNet, self-supervised, and domain-specific). Other areas of research include multi-task learning for joint classification and localization, uncertainty estimation to measure how sure a prediction is, and multimodal frameworks that combine CXR images with electronic health record data.

Although qualitative visualizations indicated anatomically plausible attention patterns, quantitative overlap analysis was not possible due to the absence of pixel-level annotations. A quantitative localization evaluation (e.g., Dice Similarity Coefficient) was therefore not performed, as the Lung X-Ray Image dataset provides only image-level labels without expert segmentation masks. As a result, direct spatial comparison between attention maps and ground-truth pathological regions could not be conducted in the current study. Future work will incorporate expert-annotated ROIs or publicly available segmentation datasets to enable more rigorous quantitative validation of attention alignment. Furthermore, clinical validation through radiologist assessment was beyond the scope of this study and remains an important direction for future collaborative investigations.

## 7 Conclusion

This study introduced HyRA-CXR, a hybrid residual–attention deep learning architecture aimed at enhancing automated chest X-ray classification. The model performs well while being lightweight, with only 0.52M parameters. This means it could be used in environments with limited computing power. The model attained average accuracy of 90.39% in extensive five-fold cross-validation and comparative evaluation, outperforming DenseNet121, Xception, and MobileNetV3-Large, thereby demonstrating generalization and stability. The ablation analysis further substantiated that both the residual and attention modules enhance diagnostic performance. In the future, we will work on adding HyRA-CXR to interactive diagnostic systems, using explainable AI (XAI) techniques for visual interpretation, and expanding the framework to include multimodal learning that combines CXR data with CT or MRI imaging.

## Data Availability

The original contributions presented in the study are included in the article/supplementary material, further inquiries can be directed to the corresponding author.
